# Arbovirus co-circulation and epidemiological convergence in a changing climate: challenges for surveillance, diagnosis and public health preparedness

**DOI:** 10.1007/s42770-026-02083-8

**Published:** 2026-09-09

**Authors:** Gian Marco Ludovici, Alba Iannotti, Colomba Russo, Francesco Gargallo di Castel Lentini, Manousos E. Kambouris, Mostafa Mohammed Atiyah, Sijo Asokan, Andrea Malizia, Marta Giovanetti

**Affiliations:** 1 Department of Biomedicine and Prevention, University of Rome Tor Vergata, Via Montpellier, 1, Rome, 00133 Italy; 2 Department of Industrial Engineering, School of Medicine and Surgery, International Master Courses in Protection Against CBRNe events, University of Rome Tor Vergata, Rome, 00133 Italy; 3 Department of Industrial Engineering, University of Rome Tor Vergata, Rome, 00133 Italy; 4 Department of Law, University of Rome Tor Vergata, Rome, 00133 Italy; 5 Department of Pharmacy, University of Patras, Patras, 26504 Greece; 6 School of Biosciences, Mar Athanasios College for Advanced Studies (MACFAST- Autonomous), Pathanamthitta, 689101 Kerala India; 7 Department of Science and Bio-Technology, Campus Bio-Medico University of Rome, Rome, 00128 Italy; 8 Laboratório de Arbovírus Hemorrágicos, Instituto Oswaldo Cruz, Fundação Oswaldo Cruz, Rio de Janeiro, 21040-900 Brazil

**Keywords:** Arboviruses, Epidemiology, Co-circulation, Surveillance systems, Public health, Climate change

## Abstract

Arboviral diseases are undergoing substantial epidemiological changes driven by climate change, urbanization, global mobility, vector expansion, and evolving ecological conditions. These processes increasingly favor the geographic overlap and co-circulation of multiple arboviruses, including in regions previously considered at limited risk. In this review, we describe this broader process as epidemiological convergence, referring to the progressive overlap of arboviral transmission systems across shared geographic, ecological, vector, host, and public health contexts. Such convergence creates interconnected challenges for clinical recognition, laboratory diagnosis, surveillance, and outbreak response. Diagnostic uncertainty is further amplified by overlapping clinical presentations, serological cross-reactivity, previous arboviral exposure, and co-infections or sequential infections. At the same time, pathogen-specific surveillance systems may inadequately capture increasingly complex transmission patterns, highlighting the need for integrated and interoperable approaches. One Health provides an essential framework for integrating human, animal, vector, and environmental dimensions of arboviral transmission. Complementarily, selected principles derived from Chemical, Biological, Radiological, Nuclear, and Explosive (CBRNE) preparedness may strengthen operational coordination, contingency planning, biosafety, risk communication, and response capacity during complex arboviral scenarios. However, their added value in arboviral settings requires further empirical evaluation. Addressing epidemiological convergence will ultimately require adaptable systems linking epidemiological understanding, diagnostic capacity, integrated surveillance, and operational preparedness.

## Introduction

Arboviruses represent a growing global health challenge, with increasing incidence and expanding geographic distribution observed over recent decades [1, 2]. Transmitted primarily by arthropod vectors such as mosquitoes, these viruses comprise a diverse group of pathogens responsible for a wide spectrum of clinical manifestations, ranging from mild febrile illness to severe neurological and hemorrhagic disease. Collectively, they impose a substantial and evolving burden on public health systems worldwide [3–8].

Recent years have been marked by significant shifts in the epidemiology of arboviral diseases. Environmental and societal factors, including climate change, urbanization, population growth, and increased global mobility, have facilitated the expansion of vector habitats and the introduction of viruses into new ecological niches. Consequently, arboviral transmission is no longer confined to traditionally endemic regions but is increasingly reported in temperate areas, including parts of Europe and North America. This transition is reshaping the global distribution of these diseases and poses new challenges for surveillance and preparedness [9, 10].

A defining feature of this evolving landscape is the co-circulation of multiple arboviruses within the same geographic regions [11]. In several settings, different arboviruses are transmitted simultaneously, often sharing vectors and affected populations [12]. This overlap increases the complexity of transmission dynamics and complicates both clinical recognition and laboratory diagnosis. Similar clinical presentations, combined with serological cross-reactivity, can lead to misclassification or underestimation of cases, particularly in resource-limited settings or during concurrent outbreaks [13, 14].

In this review, the term “epidemiological convergence” refers to the progressive overlap of previously distinct arboviral transmission systems within shared geographic, ecological, vector, host, and public health contexts. This concept extends beyond simple co-circulation, encompassing the interaction of multiple transmission cycles, overlapping vector and host populations, shared environmental drivers, and the resulting convergence of diagnostic, surveillance, and response challenges. Co-circulation can therefore be considered one of the principal observable manifestations of this broader epidemiological process [11–14].

Current surveillance systems are frequently designed to monitor individual pathogens, which may limit their capacity to capture the broader dynamics of co-circulating arboviruses [12, 14]. Fragmented surveillance approaches, together with variability in diagnostic capacity, contribute to delays in outbreak detection and challenges in accurately assessing disease burden. These limitations are especially relevant in regions experiencing the emergence or re-emergence of multiple arboviruses [2, 15, 16]. However, important uncertainties remain regarding the extent to which integrated surveillance approaches improve early detection and response across different epidemiological settings, particularly where diagnostic resources, vector monitoring capacity, and data interoperability are limited [15, 16].

Despite the increasing complexity of arboviral epidemiology, public health responses remain largely pathogen-specific, frequently focusing on single-virus outbreaks rather than integrated approaches that reflect real-world transmission patterns. This may reduce the effectiveness of interventions, particularly in contexts where multiple arboviruses co-exist and interact within the same ecological and epidemiological systems [2, 17].

This review examines the evolving epidemiology of arboviruses and explores its implications for surveillance, diagnosis, and public health response. By adopting an integrated perspective, it aims to critically examine current knowledge gaps and unresolved challenges associated with arbovirus co-circulation and epidemiological convergence, while identifying priorities for more coordinated and adaptive surveillance, diagnostic, and preparedness strategies.

## Methods

A structured literature search was conducted to identify studies relevant to the epidemiology, transmission dynamics, diagnosis, surveillance, and preparedness of arboviral diseases. Searches were performed in PubMed, Scopus, and Web of Science between January and March 2026 using combinations of the keywords “arbovirus”, “arboviral diseases”, “co-circulation”, “surveillance”, “diagnosis”, “climate change”, “vector-borne diseases”, “genomic surveillance”, “syndromic surveillance”, and “One Health”. No predefined publication-date restriction was applied, allowing the inclusion of earlier studies considered relevant for establishing the biological and epidemiological background of arboviral transmission, while greater emphasis was placed on recent evidence describing emerging transmission patterns, co-circulation, surveillance, and preparedness.

Additional information was obtained from reports and surveillance documents issued by international public health organizations, including the World Health Organization (WHO), the European Centre for Disease Prevention and Control (ECDC), the European Food Safety Authority (EFSA), and the Pan American Health Organization (PAHO).

Studies were considered eligible if they addressed one or more of the following topics: arbovirus epidemiology, vector ecology, transmission dynamics, co-circulation, diagnostic approaches, surveillance systems, genomic epidemiology, outbreak preparedness, or public health response. Original research articles, review articles, surveillance reports, and relevant public health documents published in English were included. Eligible records were evaluated according to their relevance to the predefined thematic domains of the review, including arbovirus biology and epidemiology, transmission and vector ecology, co-circulation, diagnostic challenges, surveillance strategies, and public health preparedness. Priority was given to studies providing epidemiological, microbiological, genomic, diagnostic, or surveillance evidence directly contributing to the objectives of the review.

Records identified through database searches were screened based on title and abstract relevance. Duplicate records were removed prior to screening. Full-text articles were assessed for eligibility according to their relevance to the objectives of the review. Publications not directly related to arboviruses, studies lacking sufficient epidemiological or microbiological relevance, overlapping or redundant publications, and non-English articles were excluded. At the title and abstract screening stage, 191 records were excluded because they did not address the predefined thematic scope of the review. Of the 98 full-text articles subsequently assessed for eligibility, 31 were excluded: 12 were not directly related to arboviruses, 11 fell outside the specific scope of the review, and 8 provided insufficient epidemiological relevance to the objectives of the synthesis. A total of 67 studies and documents were therefore retained for the final qualitative synthesis.

**Fig. 1:**
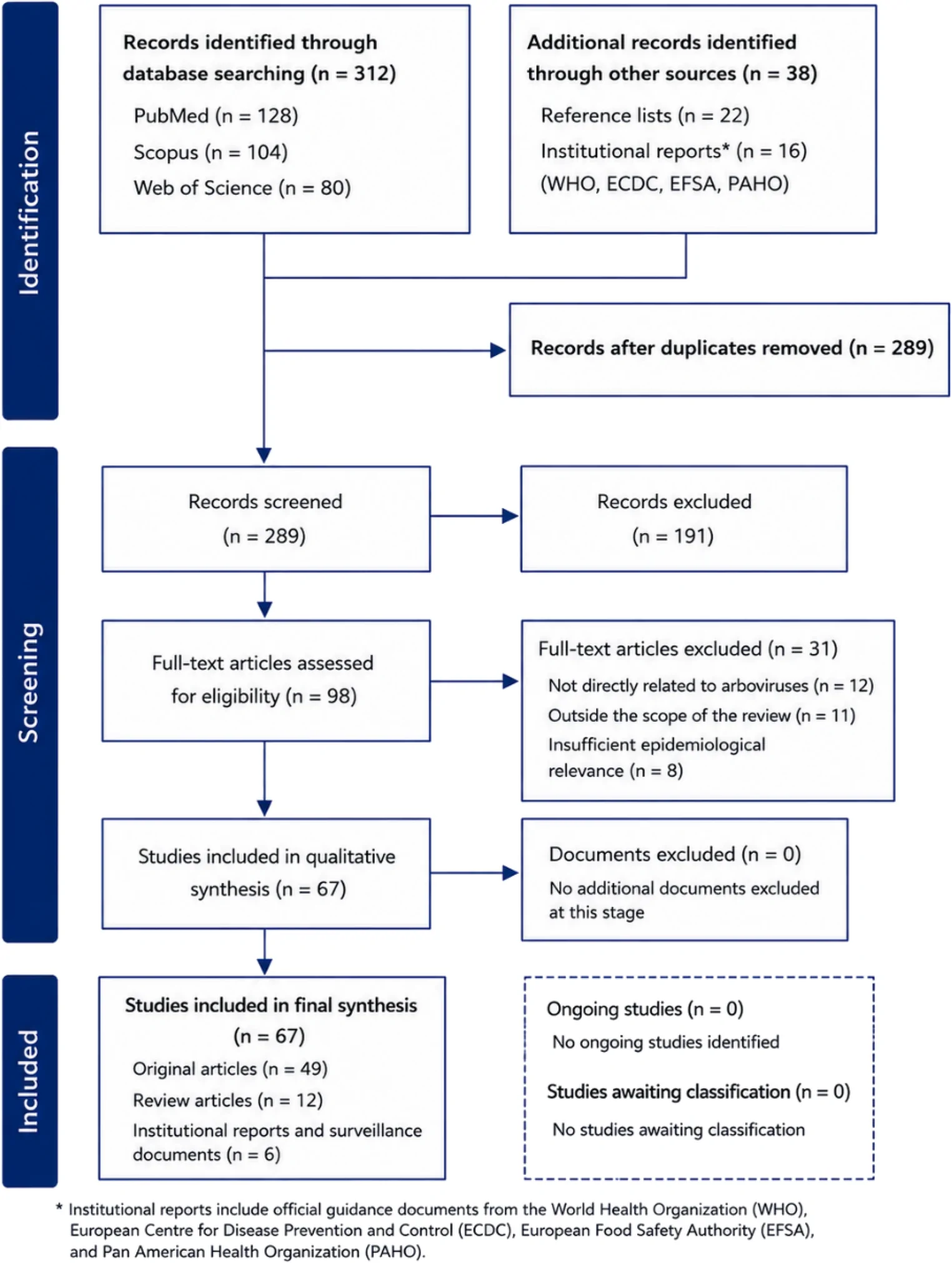
PRISMA flow diagram

The complete literature identification, screening, eligibility assessment, and inclusion process is summarized in Fig. 1.

## Arbovirus diversity and classification

Arboviruses are predominantly single-stranded RNA viruses with positive-sense genomes, although their genomic organization and replication strategies vary across viral families. Members of the *Flaviviridae* family typically possess a non-segmented RNA genome of approximately 10–11 kilobases, encoding a single polyprotein that is co- and post-translationally processed into structural and non-structural proteins [18–20]. In contrast, alphaviruses within the *Togaviridae* family have slightly larger genomes (approximately 11–12 kilobases), organized into two open reading frames that separately encode non-structural and structural proteins. These viruses are enveloped, exhibit icosahedral symmetry, and replicate in the cytoplasm following entry mediated by receptor binding and endocytosis [21, 22].

Despite these structural differences, arboviruses share key biological features, including high mutation rates driven by RNA-dependent RNA polymerases and a strong capacity for adaptation to both vertebrate hosts and arthropod vectors. This evolutionary plasticity facilitates efficient transmission across diverse ecological settings and underpins their ability to emerge and re-emerge in new geographic regions [23–25].

Arboviruses constitute a heterogeneous group spanning multiple taxonomic families and genera, unified by their transmission through arthropod vectors—primarily mosquitoes. From a virological and public health perspective, many of the most extensively studied arboviruses belong to the families *Flaviviridae* and *Togaviridae* [26–28], although clinically and epidemiologically relevant arboviruses are also found in other families, including *Peribunyaviridae*. Within *Flaviviridae*, the genus *Flavivirus* includes major human pathogens such as dengue virus (DENV), Zika virus (ZIKV), yellow fever virus (YFV), West Nile virus (WNV), Japanese encephalitis virus (JEV), and Usutu virus (USUV) [29–34]. Similarly, within *Togaviridae*, the genus *Alphavirus* encompasses viruses associated with epidemic transmission and significant morbidity, including chikungunya virus (CHIKV), Mayaro virus (MAYV), Ross River virus (RRV), Barmah Forest virus (BFV), and O’nyong-nyong virus (ONNV) [35–38]. Oropouche virus (OROV), belonging to the family Peribunyaviridae and genus Orthobunyavirus, represents another epidemiologically relevant arbovirus and illustrates the broader taxonomic and ecological diversity of this group. Its transmission cycle differs from those of many major mosquito-borne arboviruses, involving biting midges of the genus *Culicoides* as important vectors, in addition to mosquitoes in specific transmission cycles. OROV is associated primarily with acute febrile disease, although neurological manifestations may also occur [39].

Arboviruses are maintained in complex transmission cycles involving arthropod vectors and vertebrate hosts, with humans acting either as incidental hosts or as amplification hosts, depending on the specific virus and ecological context. The diversity of vectors, reservoirs, and transmission cycles contributes to the wide variability in geographic distribution and epidemiological patterns observed across arboviruses (Table 1) [39, 40].

**Table 1:** Classification and main characteristics of selected arboviruses

Virus family	Genus	Representative viruses	Transmission vector	Geographic distribution	Clinical relevance	References
*Flaviviridae*	Flavivirus	Dengue virus (DENV), Zika virus (ZIKV), Yellow fever virus (YFV)	*Aedes* mosquitoes	Tropical and subtropical regions (expanding globally)	Dengue fever, Zika syndrome, hemorrhagic disease, congenital infections	[41]
*Flaviviridae*	Flavivirus	West Nile virus (WNV), Usutu virus (USUV)	*Culex* mosquitoes	Europe, Africa, Americas, Middle East	Neuroinvasive disease, febrile illness	[42]
*Flaviviridae*	Flavivirus	Japanese encephalitis virus (JEV)	*Culex* mosquitoes	Asia and Western Pacific	Severe encephalitis	[43]
*Togaviridae*	Alphavirus	Chikungunya virus (CHIKV)	*Aedes* mosquitoes	Africa, Asia, Americas, Europe (sporadic)	Acute febrile illness, chronic arthralgia	[38]
*Togaviridae*	Alphavirus	Mayaro virus (MAYV), O’nyong-nyong virus (ONNV)	*Aedes*/*Anopheles* mosquitoes	South America (MAYV), Africa (ONNV)	Febrile illness with arthralgia	[44]
*Togaviridae*	Alphavirus	Ross River virus (RRV), Barmah Forest virus (BFV)	*Aedes/Culex* mosquitoes	Australia and Pacific regions	Polyarthritis, rash	[40]
*Peribunyaviridae*	Orthobunyavirus	Oropouche virus (OROV)	Culicoides midges, mosquitoes	South and Central America	Febrile illness, neurological involvement	[45]

The co-circulation of multiple arboviruses within overlapping ecological niches reflects the adaptability of both viruses and their vectors. In particular, the expansion of *Aedes* and *Culex* mosquito populations has facilitated the persistence and simultaneous circulation of several arboviruses within the same geographic regions [12, 14]. At the same time, the involvement of alternative arthropod vectors, such as *Culicoides* midges in OROV transmission, demonstrates that contemporary arboviral emergence cannot be interpreted exclusively through *Aedes*- and *Culex*-associated transmission models. This ecological overlap represents a key driver of the increasing complexity of arboviral epidemiology and provides the foundation for the co-circulation dynamics discussed in subsequent sections.

## Changing epidemiology of arboviruses

The epidemiology of arboviral diseases has undergone substantial changes over recent decades, driven by a combination of environmental, demographic, and socio-economic factors. Climate change has played an important role by modifying the geographic range and seasonal activity of arthropod vectors, including *Aedes* and *Culex* mosquitoes. Temperature can influence several components of vectorial capacity, including vector development and survival, biting activity, viral replication within the vector, and the duration of the extrinsic incubation period. Changes in precipitation and humidity may additionally modify the availability and persistence of breeding habitats, although their effects can vary according to vector species and local ecological conditions. Together, these mechanisms may extend transmission seasons and increase the suitability of regions previously considered less favorable for sustained arboviral transmission [2, 9, 10]. Importantly, climate-sensitive changes in arboviral transmission are not restricted to mosquito-borne systems, as the involvement of other arthropod vectors, including *Culicoides* midges, broadens the range of ecological responses that may influence emerging transmission patterns.

In parallel, rapid urbanization and population growth have contributed to the creation of densely populated environments that facilitate vector breeding and virus transmission. Informal settlements, inadequate water management, and increased human–vector contact represent key drivers of arboviral spread, particularly in low- and middle-income settings [46, 47].

Global mobility has further accelerated the dissemination of arboviruses. International travel and trade enable the rapid movement of infected individuals and vectors across continents, contributing to the introduction of viruses into new regions. This process has been particularly evident in the emergence of arboviral transmission in temperate areas, including Europe and North America [9, 10, 48, 49].

Recent epidemiological data illustrate the scale and complexity of this expansion. Large outbreaks continue to occur across multiple continents, often involving substantial case numbers and, in some instances, significant mortality. For example, CHIKV has shown marked resurgence in recent years, with more than 1.6 million suspected cases reported globally during 2024–2025, including major outbreaks in Asia and increasing transmission in Europe. Similarly, dengue remains one of the most widespread arboviral diseases globally, with recurrent epidemics placing considerable strain on health systems [2, 10, 50–52].

A defining feature of the current epidemiological landscape is the co-circulation of multiple arboviruses within the same regions. In several settings, different viruses are transmitted by shared vector populations, resulting in overlapping outbreaks and increased complexity in transmission dynamics. This pattern challenges traditional epidemiological models, which often consider arboviral diseases in isolation [37, 49, 53].

The expansion of arboviruses into new geographic areas, together with the increasing frequency of outbreaks, highlights the limitations of existing surveillance systems and preparedness strategies. As transmission patterns continue to evolve, there is a growing need for approaches that account for the interconnected nature of arboviral diseases and their shared ecological drivers [9, 27, 37, 51].

However, the relationship between environmental change and arboviral emergence is not uniform across viruses, vectors, or geographic settings. The relative contribution of climate, vector adaptation, urbanization, human mobility, and local ecological conditions remains difficult to disentangle, limiting the generalizability of predictive models across different transmission systems [9, 10, 27].

## Co-circulation and transmission complexity

The co-circulation of multiple arboviruses represents a defining feature of the current epidemiological landscape. Rather than occurring in isolation, arboviral transmission increasingly involves overlapping viral circulation within shared ecological and vector systems, resulting in more complex and dynamic outbreak patterns [12, 28, 54].

This phenomenon is largely driven by the widespread distribution of competent vectors, particularly *Aedes* and *Culex* mosquito species, which are capable of transmitting multiple viruses within the same environment [51, 55]. In tropical and subtropical regions, several arboviruses may circulate simultaneously within the same human populations. In temperate areas, the expansion of vector habitats has enabled the establishment of seasonal transmission cycles involving multiple viruses, particularly those associated with *Culex* species [56–60].

The co-circulation of multiple arboviruses within shared ecological niches alters traditional transmission dynamics. Vector competence for different viruses, together with environmental variables such as temperature, rainfall, and humidity, influences viral amplification and seasonal patterns. In addition, enzootic cycles involving animal reservoirs contribute to viral persistence and may act as sources of re-emergence, further complicating transmission pathways [2, 12, 53].

When competent vectors are exposed to more than one arbovirus, interactions between viruses within the vector may further influence transmission efficiency. Such interactions may be neutral, competitive, or potentially facilitative, depending on the viruses involved, the vector species, infection timing, and environmental conditions. Consequently, the presence of multiple arboviruses within the same ecological system does not necessarily imply equivalent transmission potential for each virus, and important uncertainties remain regarding how vector-level interactions translate into population-level transmission patterns [12, 28, 54, 55].

From a clinical perspective, co-circulation introduces significant challenges for disease recognition. Arboviral infections frequently present with overlapping and non-specific symptoms, including fever, rash, arthralgia, and, in some cases, neurological manifestations. This similarity in clinical presentation makes differentiation difficult, particularly during periods of increased transmission or in regions experiencing concurrent outbreaks [61, 62].

At the population level, co-circulation may also lead to co-infections or sequential infections, although these are not always systematically detected. Such interactions may influence disease severity, immune response, and transmission dynamics, although their full impact remains incompletely understood. The presence of multiple viruses within the same transmission system therefore represents not only an epidemiological challenge but also a biological one [12, 28, 54].

Recent epidemiological patterns further highlight the significance of this phenomenon. In several regions, overlapping transmission of arboviruses has been documented, reflecting the increasing convergence of ecological and environmental drivers [49, 57]. In Europe, for example, the simultaneous circulation of West Nile and Usutu viruses illustrates how multiple flaviviruses can establish parallel transmission cycles within the same vector populations [56]. In tropical regions, concurrent transmission of different arboviruses has been associated with increased diagnostic uncertainty and challenges in outbreak management [41, 42, 63].

The shift from single-pathogen transmission models to multi-pathogen systems has important implications for both surveillance and response. Approaches focused on individual viruses may fail to capture the full extent of transmission dynamics, leading to incomplete assessments of disease burden and delayed outbreak detection [28, 37].

Overall, the co-circulation of arboviruses reflects a broader transition toward more interconnected and dynamic epidemiological systems. Understanding these interactions is essential to improve the interpretation of surveillance data, refine diagnostic strategies, and support the development of more effective and integrated public health responses [28, 37, 64].

In regions characterized by the simultaneous circulation of multiple arboviruses, co-infections and sequential infections are increasingly recognized as important but still under-investigated phenomena. Concurrent infections with DENV, CHIKV and ZIKV viruses have been documented in several endemic settings and may complicate both clinical management and laboratory diagnosis [2, 14, 54]. Moreover, sequential infections may modify subsequent immune responses through pre-existing cross-reactive immunity, potentially influencing both disease severity and the interpretation of serological findings. However, these effects are virus- and context-dependent, and current evidence does not support uniform assumptions regarding whether prior arboviral exposure results in protection, enhanced disease, or limited clinical impact. Further research is therefore required to clarify the immunological, epidemiological, and clinical consequences of sequential infections and their implications for surveillance and disease burden assessment [12, 14, 54].

## Diagnostic challenges

The diagnosis of arboviral infections remains a major challenge, particularly in settings characterized by the co-circulation of multiple viruses. Although advances in molecular and serological techniques have improved diagnostic capabilities, several limitations continue to affect the accuracy and timeliness of case identification [61, 65–67].

One of the primary challenges is the overlap in clinical presentation among arboviral diseases. Many infections present with non-specific symptoms such as fever, headache, myalgia, and, in some cases, rash or neurological involvement. These similarities limit the reliability of clinical diagnosis, particularly during periods of increased transmission or in regions where multiple arboviruses are endemic or emerging [57, 67–69].

Laboratory diagnosis is further complicated by the biological characteristics of arboviruses. Molecular methods, such as reverse transcription polymerase chain reaction (RT-PCR), provide high specificity and are considered the gold standard during the early phase of infection. However, their diagnostic window is limited, as viral RNA is detectable only during a relatively short period of viremia. Delayed presentation or late sample collection may therefore result in false-negative results [27, 66, 67].

Serological assays remain widely used due to their practicality and accessibility, particularly in resource-limited settings. However, cross-reactivity represents a major limitation, especially among viruses belonging to the same family. Antibody responses to flaviviruses, for example, often overlap substantially, making it difficult to distinguish between infections based on serology alone. This challenge is further exacerbated in populations with prior arboviral exposure or vaccination, where pre-existing and cross-reactive antibodies may substantially influence serological profiles and complicate the distinction between previous and recent infections [27, 57, 65–68, 70].

Beyond their diagnostic implications, pre-existing cross-reactive antibodies may also influence the biological outcome of subsequent flavivirus infections. Antibody-dependent enhancement (ADE), most extensively characterized in dengue, describes a mechanism by which non-neutralizing or sub-neutralizing antibodies may facilitate viral entry into Fc receptor-bearing cells and potentially contribute to increased disease severity. However, the relevance and magnitude of this phenomenon vary according to the virus, immune history, antibody concentration, and epidemiological context, and it should not be assumed that cross-reactive immunity uniformly results in disease enhancement [5, 25, 65, 70]. From a diagnostic perspective, these immunological interactions reinforce the need to interpret serological results in relation to vaccination history, previous arboviral exposure, timing of sample collection, and the local epidemiological context [65, 67, 70].

In addition, variability in diagnostic performance across assays and settings contributes to uncertainty in case classification. Rapid diagnostic tests, while useful for point-of-care screening, often show reduced sensitivity in early infection and may lack validation across different viral lineages. More advanced approaches, including multiplex molecular assays, offer the potential to detect multiple pathogens simultaneously. However, their availability remains limited, and implementation may be constrained by cost and infrastructure requirements [27, 67, 68, 70].

The increasing complexity of arboviral epidemiology further amplifies these diagnostic challenges. Co-infections and sequential infections, although not always systematically documented, may occur in areas of intense transmission, complicating both clinical interpretation and laboratory confirmation. In such contexts, reliance on single-pathogen diagnostic approaches may lead to incomplete or inaccurate assessment of disease burden [27, 57, 65–68, 70].

From a public health perspective, these limitations have important implications. Delayed or inaccurate diagnosis can affect surveillance data, hinder outbreak detection, and complicate the implementation of targeted interventions. Strengthening diagnostic capacity therefore represents a critical component of effective arbovirus control, requiring improvements in laboratory infrastructure, standardization of diagnostic protocols, and broader access to sensitive and specific testing methods [27, 37, 64].

Nevertheless, no single diagnostic approach can fully resolve the challenges posed by co-circulating arboviruses. Molecular, serological, and multiplex methods provide complementary information but remain dependent on infection timing, local epidemiology, laboratory capacity, and appropriate interpretation. Future diagnostic strategies should therefore prioritize integrated algorithms rather than reliance on individual assays, particularly in settings characterized by simultaneous or sequential arboviral exposure [27, 65, 67, 70].

## Surveillance gaps and outbreak dynamics

The increasing complexity of arboviral epidemiology has exposed significant limitations in current surveillance systems. Most frameworks remain pathogen-specific, designed to monitor individual viruses rather than capturing the broader dynamics of co-circulating arboviruses. This fragmentation can result in incomplete datasets, delayed outbreak detection, and challenges in coordinating timely public health responses [27, 64]. Recent epidemiological data from major arboviral outbreaks further illustrate the scale, variability, and geographic expansion of these infections (Table 2).

**Table 2:** Selected recent arboviral outbreaks and epidemiological characteristics

Virus	Region / Country	Period	Reported cases	Deaths	References
Dengue virus	Latin America and Caribbean	2024	13,671,322 suspected cases	8,186 deaths	[71]
Chikungunya virus	Global	2023–2025	1,600,000 (estimated)	155 deaths	[72]
Chikungunya virus	China (Guangdong)	2025	160,000 (reported)	Not reported	[73]
Chikungunya virus	Europe (France, Italy, Réunion, Mayotte)	2025	56,456 cases	40 deaths	[74]
West Nile virus	Europe	2025	1,133 locally acquired cases	92 deaths	[75]
West Nile virus	Italy	2025	332 confirmed cases	27 deaths	[76]
Zika virus	Americas	2015–2017 (peak epidemic)	Approximately 800,000 suspected cases (Brazil alone)	Not systematically reported	[77]
Usutu virus	Europe	2018–2025	Sporadic human cases (< 100/year)	Low reported mortality	[78]

A main challenge lies in the heterogeneity of surveillance infrastructures across regions. Differences in laboratory capacity, reporting systems, and case definitions contribute to variability in data quality and comparability. In many settings, passive surveillance remains the primary approach, relying on clinically apparent cases and thereby underestimating the true burden of disease, particularly for arboviruses with a high proportion of asymptomatic or mild infections [27, 37, 64].

Recent outbreak data highlight the scale and variability of arboviral transmission. Dengue, for example, remains the most widespread arboviral disease globally, with unprecedented epidemic activity in recent years [41, 79]. The 2024 outbreak in Latin America and the Caribbean exceeded 13 million suspected cases and more than 8,000 deaths, representing one of the largest arboviral epidemics recorded. This magnitude illustrates the capacity of arboviruses to generate large-scale outbreaks capable of overwhelming health systems, particularly when surveillance and response mechanisms are not adequately integrated [61, 80, 81].

In parallel, arboviral epidemiology in temperate regions has evolved substantially. West Nile virus has become established across large parts of Europe, with seasonal transmission patterns shaped by environmental conditions and vector dynamics. Surveillance data from 2025 indicate widespread circulation across multiple countries, with human cases reported in numerous regions [27, 37, 80]. Italy provides a particularly relevant example of this transition. Recent data indicate a substantial increase in West Nile virus activity, with hundreds of confirmed cases and associated fatalities reported during the 2025 transmission season, particularly in central and southern regions. Importantly, the geographic distribution of cases has expanded beyond historically affected areas, reflecting a shift from localized outbreaks to more widespread and established transmission [76, 82]. Recent observations also suggest changes in the timing and duration of seasonal transmission, further highlighting the potential influence of climatic and ecological drivers. These observations illustrate how arboviral outbreaks are no longer confined to specific geographic or temporal boundaries but are increasingly characterized by dynamic and overlapping transmission patterns. The expansion of arboviruses into new regions, combined with the persistence of endemic transmission in others, creates a complex and interconnected epidemiological landscape [27, 80].

Another critical issue is the limited integration of human, animal, and vector surveillance. In recent years, genomic surveillance has become an essential component of arbovirus monitoring. Whole-genome sequencing and phylogenetic analyses provide valuable information regarding viral evolution, introduction events, transmission chains, and geographic spread. These approaches have significantly improved the understanding of outbreaks involving dengue, chikungunya, Zika, yellow fever, and West Nile viruses [83, 84]. Integrating genomic data with traditional epidemiological surveillance may enhance outbreak detection and support more targeted public health interventions [28].

However, the effectiveness of genomic surveillance remains strongly dependent on representative sampling, sequencing capacity, timely data generation, and integration with epidemiological and entomological information. Uneven genomic coverage across regions may therefore limit the interpretation of transmission patterns and reduce the generalizability of genomic inferences, particularly in settings with limited laboratory infrastructure [28, 31, 64].

Syndromic surveillance approaches may also contribute to earlier detection of arboviral activity, particularly in regions where laboratory confirmation is limited [85]. Monitoring clusters of febrile illness, neurological syndromes, or other clinical indicators can provide early warning signals before pathogen-specific diagnoses become available. Although syndromic systems lack specificity, their integration with laboratory and genomic surveillance may improve situational awareness and outbreak preparedness [64].

Many arboviruses circulate through zoonotic cycles involving animal reservoirs, yet surveillance systems are often not fully coordinated across sectors. The lack of integrated “One Health” approaches may delay the detection of early signals of viral circulation, particularly in enzootic phases preceding human outbreaks [51, 86, 87].

In addition, real-time data sharing remains a challenge. Delays in reporting, inconsistencies in data availability, and limited interoperability between surveillance platforms can hinder timely decision-making. This is particularly relevant during rapidly evolving outbreaks, where early detection and rapid response are essential to limit transmission [64, 82, 88].

Taken together, these limitations highlight the need for more integrated and adaptive surveillance systems capable of capturing the complexity of arboviral transmission [88]. Strengthening surveillance requires not only improved diagnostic capacity and data collection but also enhanced coordination across sectors and geographic regions. In an increasingly interconnected epidemiological environment, surveillance systems must evolve from pathogen-specific models toward more comprehensive approaches that reflect the reality of co-circulating arboviruses [64, 82, 88].

Importantly, greater integration does not automatically translate into more effective surveillance. The added value of integrated systems depends on data quality, interoperability, sustained laboratory and entomological capacity, and the ability to translate heterogeneous surveillance signals into timely public health action. Future efforts should therefore focus not only on expanding data collection but also on evaluating which combinations of epidemiological, genomic, syndromic, animal, and vector surveillance provide actionable information across different epidemiological settings [64, 82, 88].

## Applying preparedness principles to complex arboviral surveillance scenarios

The evolving epidemiology of arboviral diseases highlights the need for more coordinated and adaptive response strategies. The increasing co-circulation of multiple arboviruses, together with the expansion of transmission into new geographic areas, challenges traditional public health approaches that are often structured around single-pathogen surveillance and response models. Such scenarios require not only integrated surveillance but also the capacity to translate epidemiological signals into coordinated operational responses across laboratories, healthcare services, vector-control programmes, and public health authorities [12, 14, 37, 80].

In this context, there is growing recognition that arboviral outbreaks should be approached within broader frameworks of emergency preparedness [89–92]. Although arboviral diseases are not traditionally classified as Chemical, Biological, Radiological, Nuclear, and Explosive (CBRNE) events, the increasing convergence of multiple arboviruses, their transboundary spread, the growing pressure on surveillance systems, and the need for rapid multi-sectoral coordination share several operational characteristics with complex biological threats. Consequently, frameworks conceptually aligned with CBRNE preparedness may offer a useful perspective for integrating surveillance, biosafety, risk communication, diagnostics, and response activities within a coordinated operational model. Rather than replacing established public health systems, these frameworks may complement existing approaches by strengthening preparedness, interoperability, and resilience in scenarios characterized by overlapping outbreaks and increasing epidemiological complexity [93–95].

The proposed contribution of CBRNE-derived preparedness principles should be distinguished from, rather than positioned as an alternative to, the One Health approach. One Health provides an essential framework for understanding and addressing the interdependence of human, animal, and environmental health, which is particularly relevant to arboviruses maintained through complex vector–host transmission cycles [86, 87]. CBRNE-derived preparedness principles address a different, predominantly operational dimension, emphasizing contingency planning, defined coordination structures, interoperability, biosafety, risk communication, resource mobilization, and continuity of essential services during complex biological emergencies [89–96]. In this sense, One Health can support the identification and integration of epidemiological signals across sectors, whereas preparedness-oriented principles may help translate those signals into coordinated actions when rapid escalation and multi-agency response are required. The two perspectives should therefore be regarded as complementary rather than competing frameworks.

Within this complementary framework, arboviral preparedness should prioritize the development of surveillance systems capable of capturing signals across multiple pathogens simultaneously. This requires improved data integration, interoperability between surveillance platforms, and closer alignment between human, animal, and vector monitoring systems within a One Health perspective. Early identification of transmission patterns, including enzootic circulation, is essential to prevent progression to large-scale outbreaks [64, 82, 88, 91, 94]. The operational value of preparedness-oriented frameworks becomes particularly evident in regions experiencing simultaneous circulation of multiple arboviruses, where surveillance activities, laboratory diagnostics, vector control programmes, healthcare services, and public communication strategies must be coordinated across different sectors and administrative levels [64, 96].

Their potential added value is greatest when routine surveillance must transition rapidly into emergency response—for example, when concurrent transmission generates diagnostic uncertainty, laboratory demand exceeds routine capacity, vector-control activities require rapid geographical expansion, or healthcare and public communication systems must respond simultaneously. Under these conditions, predefined responsibilities, escalation criteria, communication pathways, and contingency procedures may reduce fragmentation between surveillance and response activities [16, 89–96].

Diagnostic capacity represents another critical component of an integrated framework. Expanding access to molecular diagnostics, promoting the use of multiplex assays, and standardizing laboratory protocols across regions may help to address the limitations associated with cross-reactivity and delayed case confirmation. In parallel, strengthening laboratory networks and ensuring rapid data sharing can enhance situational awareness during outbreaks [64, 67, 90, 97].

The incorporation of biosafety principles within these frameworks is particularly relevant in healthcare and laboratory settings [93]. Although arboviruses are primarily vector-borne, secondary risks associated with transfusion, transplantation, and occupational exposure have been documented. In addition, the management of suspected or confirmed cases requires appropriate infection prevention measures to protect healthcare workers and maintain continuity of care. Integrating biosafety considerations into outbreak response planning can therefore contribute to reducing secondary transmission risks and improving overall system resilience [91, 94, 98].

From an operational perspective, the objective is therefore not to create parallel response structures, but to strengthen coordination among existing public health authorities, laboratories, healthcare providers, vector-control services, and communication systems through predefined and interoperable response mechanisms [64, 90, 95].

Taken together, these elements support the development of more integrated and adaptive response frameworks that reflect the complexity of arboviral transmission. While not replacing existing public health or One Health structures, the incorporation of selected CBRNE-derived preparedness principles may strengthen their operational capacity during complex arboviral emergencies. However, their effectiveness in arboviral settings requires further evaluation, and future studies should assess whether such preparedness models measurably improve response times, intersectoral coordination, resource allocation, and outbreak outcomes [16, 37, 64, 96, 97].

## Conclusion

The contemporary epidemiology of arboviral diseases is increasingly characterized not only by geographic expansion and recurrent outbreaks, but also by epidemiological convergence across viruses, vectors, hosts, and affected populations. Co-circulation therefore represents more than the simultaneous presence of multiple arboviruses within the same geographic area; it creates interconnected biological, diagnostic, surveillance, and operational challenges that are not always adequately captured by traditional single-pathogen approaches.

Addressing this complexity requires surveillance and diagnostic strategies capable of integrating heterogeneous information while recognizing their inherent limitations. Genomic, epidemiological, syndromic, animal, and vector surveillance can provide complementary signals, but their effectiveness depends on data quality, representative sampling, interoperability, laboratory capacity, and the ability to translate information into timely public health action. Similarly, advances in molecular, serological, and multiplex diagnostics should increasingly be incorporated into context-specific diagnostic algorithms that account for co-infections, sequential exposures, vaccination history, and local patterns of arboviral circulation.

From a preparedness perspective, One Health and CBRNE-derived principles should be regarded as complementary rather than competing approaches. One Health provides the framework required to integrate human, animal, vector, and environmental dimensions of arboviral transmission, whereas selected CBRNE-derived preparedness principles may contribute to the operational coordination required when surveillance signals evolve into complex biological emergencies. Their potential contribution lies particularly in contingency planning, interoperability, biosafety, risk communication, resource mobilization, and coordination among existing response structures, rather than in the creation of parallel systems.

Important knowledge gaps nevertheless remain. Future research should clarify the biological and clinical consequences of co-infections and sequential arboviral exposures, characterize virus–vector interactions under changing environmental conditions, improve the representativeness and interoperability of integrated surveillance systems, and evaluate diagnostic algorithms in settings where multiple arboviruses co-circulate. Importantly, the operational value of preparedness models incorporating CBRNE-derived principles should also be evaluated empirically, including their effects on response times, intersectoral coordination, resource allocation, and outbreak outcomes.

Ultimately, responding effectively to the evolving arboviral landscape will require moving beyond isolated pathogen-specific perspectives toward systems capable of linking epidemiological understanding, integrated surveillance, diagnostic capacity, and operational preparedness. Such an approach may provide a more adaptable foundation for managing arboviral threats in an increasingly interconnected and environmentally dynamic world.

## Data Availability

All data generated or analyzed during this study are included in this published article. For other data, these may be requested through the corresponding author.

## Declarations

The manuscript has been read and approved by all the authors.
